# 1′-Benzyl-4,4′′-bis­(4-chloro­phen­yl)-3-(2,6-dichloro­phen­yl)-1′′-methyl-4,5-dihydro­isoxazole-5-spiro-3′-piperidine-5′-spiro-3′′-pyrrolidine-2′′-spiro-3′′′-indoline-2′′′,4′-dione

**DOI:** 10.1107/S1600536809053276

**Published:** 2009-12-16

**Authors:** Yongjiang Hou

**Affiliations:** a School of Environmental Science and Engineering, Hebei University of Science and Technology, 050018 Shijiazhuang, Hebei Province, People’s Republic of China

## Abstract

The asymmetric unit of the title compound, C_43_H_34_Cl_4_N_4_O_3_, contains two crystallographically independent mol­ecules. In both mol­ecules, the pyrrolidine ring adopts a twist conformation, the oxindole units are slightly distorted from planarity and the isoxazoline ring adopts an envelope conformation. The crystal structure is stabilized by N—H⋯O hydrogen-bonding inter­actions giving one-dimensional chain structures.

## Related literature

For the biological activity of spiro compounds, see: James *et al.* (1991 ▶); Kobayashi *et al.* (1991 ▶). For the use of 1,3-dipolar cyclo­addition reactions in the construction of spiro compounds, see: Caramella & Grunanger (1984 ▶).
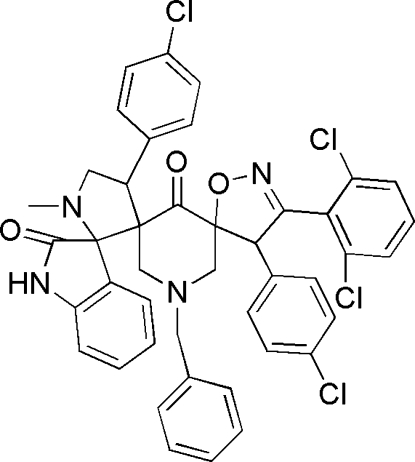


## Experimental

### 

#### Crystal data

C_43_H_34_Cl_4_N_4_O_3_
*M* *_r_* = 796.54
Orthorhombic, 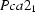

*a* = 25.588 (5) Å
*b* = 8.8028 (18) Å
*c* = 33.195 (7) Å
*V* = 7477 (3) Å^3^
*Z* = 8
Cu *K*α radiation
μ = 3.26 mm^−1^
*T* = 113 K
0.38 × 0.20 × 0.18 mm

#### Data collection

Rigaku Saturn CCD area-detector diffractometer
Absorption correction: multi-scan (*CrystalClear*; Rigaku, 2001 ▶) *T*_min_ = 0.371, *T*_max_ = 0.592
35641 measured reflections
12162 independent reflections
10126 reflections with *I* > 2σ(*I*)
*R*_int_ = 0.059

#### Refinement

*R*[*F* ^2^ > 2σ(*F* ^2^)] = 0.051
*wR*(*F* ^2^) = 0.128
*S* = 1.00
12162 reflections
982 parameters
1 restraint
H atoms treated by a mixture of independent and constrained refinement
Δρ_max_ = 0.68 e Å^−3^
Δρ_min_ = −0.65 e Å^−3^
Absolute structure: Flack (1983), 4589 Friedel pairs
Flack parameter: 0.187 (12)

### 

Data collection: *CrystalClear* (Rigaku, 2001 ▶); cell refinement: *CrystalClear*; data reduction: *CrystalClear*; program(s) used to solve structure: *SHELXS97* (Sheldrick, 2008 ▶); program(s) used to refine structure: *SHELXL97* (Sheldrick, 2008 ▶); molecular graphics: *SHELXTL* (Sheldrick, 2008 ▶); software used to prepare material for publication: *SHELXTL*.

## Supplementary Material

Crystal structure: contains datablocks I, global. DOI: 10.1107/S1600536809053276/zs2026sup1.cif

Structure factors: contains datablocks I. DOI: 10.1107/S1600536809053276/zs2026Isup2.hkl

Additional supplementary materials:  crystallographic information; 3D view; checkCIF report

## Figures and Tables

**Table 1:** Hydrogen-bond geometry (Å, °)

*D*—H⋯*A*	*D*—H	H⋯*A*	*D*⋯*A*	*D*—H⋯*A*
N4—H4⋯O6^i^	0.87 (5)	1.94 (5)	2.804 (4)	176 (4)
N8—H8⋯O3^ii^	0.68 (5)	2.14 (5)	2.808 (4)	173 (5)

Symmetry codes: (i) 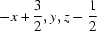; (ii) 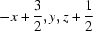.

**Fig. 1.**
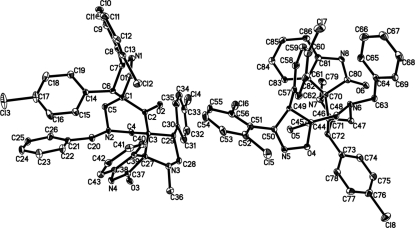
The molecular structure of (I) with the atom-labeling scheme. Ellipsoids are drawn at the 30% probability level. H atoms have been omitted for clarity.

## supplementary crystallographic information

### Comment

Spiro-compounds represent an important class of naturally occurring substances, which in many cases exhibit useful biological properties (Kobayashi *et al.*, 1991; James *et al.*, 1991). 1,3-Dipolar cycloaddition reactions are widely used for the construction of spiro-compounds (Caramella & Grunanger,1984). In this paper, the structure of the synthetic title compound C_43_H_34_Cl_4_N_4_O_3_ (I) is reported. In (I) the asymmetric unit contains two crystallographically independent spiro molecules (Fig. 1). In both molecules, the pyrrolidine ring adopts a twist conformation, the oxindole moieties are slightly distorted from planarity and the isoxazoline ring adopts an envelope conformation. The molecular structure is stabilized by intermolecular N—H···O hydrogen-bonding interactions (Table 1) giving one-dimensional chain structures. The absolute configuration for (I) was not confirmed in this analysis, the Flack parameter (Flack, 1983) [0.187 (12): 4589 Friedel pairs] for the configuration reported [C1,C44(*S*, C3,C46*R*, C6,C49*R*, C27,C70(*S*, C29,C72(*S*)] being inconclusive.

### Experimental

A mixture of 1''-Benzyl-5''-(4-chlorobenzylidene)-4'-(4-chlorophenyl)-1'-methyl-2,3-\ dihydro-1*H*-indole-3-spiro-2'-pyrrolidine-3'-spiro-3''-piperidine-2,\ 3''-dione (1 mmol) and 2,6-dichloro-benzonitrile oxide (1.5 mmol) were dissolved in dry benzene (30 ml) and heated under reflux for 24 h. After completion of the reaction as indicated by TLC, the solvent was evaporated under vacuum. The residue was purified by column chromatography employing a petroleum ether/ethyl acetate mixture (5:1 *v*/*v*) as eluent, to obtain the title compound (I). A 20 mg sample of (I) was dissolved in 15 ml of a petroleum ether-ethyl acetate mixture which was allowed to evaporate at room temperature over 15 days, giving colorless single crystals suitable for X-ray analysis.

### Refinement

All H atoms attached to C were fixed geometrically and treated as riding with C—H = 0.95 Å (aromatic), 0.98 Å (methyl), 0.99 Å (methylene), 1.00 Å (methine) with *U*_iso_(H) = 1.2*U*_eq_(C) or 1.5*U*_eq_(methane C). The H atoms on N4 and N8 were located in a difference Fourier and both positional and isotropic displacement parameters were refined. The absolute configuration for both molecules [C1, C44(*S*, C3, C46*R*, C6, C49*R*, C27, C70(*S*, C29, C72(*S*)], as indicated by the Flack parameter [0.187 (12): 4589 Friedel pairs], was not conclusive but was adopted as a better alternative to that for the enantiomer.

### Crystal data

**Table:**

C_43_H_34_Cl_4_N_4_O_3_	*D*_x_ = 1.415 Mg m^−^^3^
*M**_r_* = 796.54	Melting point: 515 K
Orthorhombic, *P**c**a*2_1_	Cu *K*α radiation, λ = 1.54187 Å
Hall symbol: P 2c -2ac	Cell parameters from 8065 reflections
*a* = 25.588 (5) Å	θ = 27.6–72.7°
*b* = 8.8028 (18) Å	µ = 3.26 mm^−^^1^
*c* = 33.195 (7) Å	*T* = 113 K
*V* = 7477 (3) Å^3^	Prism, colorless
*Z* = 8	0.38 × 0.20 × 0.18 mm
*F*(000) = 3296	

### Data collection

**Table:**

Rigaku Saturn CCD area-detector diffractometer	12162 independent reflections
Radiation source: fine-focus sealed tube	10126 reflections with *I* > 2σ(*I*)
graphite	*R*_int_ = 0.059
Detector resolution: 11.11 pixels mm^-1^	θ_max_ = 72.7°, θ_min_ = 2.7°
ω and φ scans	*h* = −31→31
Absorption correction: multi-scan (*CrystalClear*; Rigaku, 2001)	*k* = −7→10
*T*_min_ = 0.371, *T*_max_ = 0.592	*l* = −40→33
35641 measured reflections	

### Refinement

**Table:**

Refinement on *F*^2^	Secondary atom site location: difference Fourier map
Least-squares matrix: full	Hydrogen site location: inferred from neighbouring sites
*R*[*F*^2^ > 2σ(*F*^2^)] = 0.051	H atoms treated by a mixture of independent and constrained refinement
*wR*(*F*^2^) = 0.128	*w* = 1/[σ^2^(*F*_o_^2^) + (0.0763*P*)^2^] where *P* = (*F*_o_^2^ + 2*F*_c_^2^)/3
*S* = 1.00	(Δ/σ)_max_ = 0.001
12162 reflections	Δρ_max_ = 0.68 e Å^−^^3^
982 parameters	Δρ_min_ = −0.65 e Å^−^^3^
1 restraint	Absolute structure: Flack (1983), 4589 Friedel pairs
Primary atom site location: structure-invariant direct methods	Flack parameter: 0.187 (12)

### Special details

**Table:**

Geometry. All e.s.d.'s (except the e.s.d. in the dihedral angle between two l.s. planes) are estimated using the full covariance matrix. The cell e.s.d.'s are taken into account individually in the estimation of e.s.d.'s in distances, angles and torsion angles; correlations between e.s.d.'s in cell parameters are only used when they are defined by crystal symmetry. An approximate (isotropic) treatment of cell e.s.d.'s is used for estimating e.s.d.'s involving l.s. planes.
Refinement. Refinement of *F*^2^ against ALL reflections. The weighted *R*-factor *wR* and goodness of fit *S* are based on *F*^2^, conventional *R*-factors *R* are based on *F*, with *F* set to zero for negative *F*^2^. The threshold expression of *F*^2^ > σ(*F*^2^) is used only for calculating *R*-factors(gt) *etc*. and is not relevant to the choice of reflections for refinement. *R*-factors based on *F*^2^ are statistically about twice as large as those based on *F*, and *R*- factors based on ALL data will be even larger.

### Fractional atomic coordinates and isotropic or equivalent isotropic displacement parameters (Å^2^)

**Table:**

	*x*	*y*	*z*	*U*_iso_*/*U*_eq_	
Cl1	0.40006 (4)	1.32448 (11)	0.48020 (3)	0.0316 (2)	
Cl2	0.43932 (4)	0.71264 (10)	0.47298 (3)	0.0312 (2)	
Cl3	0.32260 (6)	1.08502 (16)	0.28205 (4)	0.0699 (5)	
Cl4	0.80487 (5)	1.45614 (14)	0.43692 (4)	0.0504 (3)	
Cl5	0.65692 (4)	0.15562 (12)	0.54840 (3)	0.0357 (2)	
Cl6	0.69754 (4)	0.76332 (11)	0.53592 (3)	0.0358 (2)	
Cl7	0.58501 (5)	0.43025 (14)	0.74233 (4)	0.0507 (3)	
Cl8	1.06625 (5)	0.06761 (15)	0.58627 (4)	0.0514 (3)	
N1	0.49888 (11)	1.1480 (3)	0.46582 (8)	0.0230 (6)	
N2	0.57768 (10)	1.0529 (3)	0.33825 (8)	0.0195 (6)	
N3	0.65828 (12)	0.6533 (4)	0.38483 (9)	0.0260 (7)	
N4	0.58478 (12)	0.6975 (4)	0.30111 (9)	0.0242 (6)	
H4	0.5815 (17)	0.697 (5)	0.2750 (14)	0.029*	
N5	0.75677 (11)	0.3436 (3)	0.55589 (8)	0.0222 (6)	
N6	0.83536 (11)	0.4556 (4)	0.68193 (9)	0.0223 (6)	
N7	0.91228 (12)	0.8600 (4)	0.63442 (9)	0.0247 (7)	
N8	0.83980 (13)	0.8136 (4)	0.71811 (9)	0.0252 (7)	
H8	0.8372 (18)	0.820 (5)	0.7383 (15)	0.030*	
O1	0.53832 (9)	1.1680 (3)	0.43659 (7)	0.0216 (5)	
O2	0.57444 (9)	0.8760 (3)	0.44919 (7)	0.0248 (5)	
O3	0.66436 (10)	0.8166 (3)	0.30265 (7)	0.0270 (6)	
O4	0.79718 (9)	0.3346 (3)	0.58406 (7)	0.0230 (5)	
O5	0.83066 (10)	0.6288 (3)	0.57014 (7)	0.0262 (6)	
O6	0.91991 (10)	0.6987 (3)	0.71672 (7)	0.0271 (6)	
C1	0.53390 (13)	1.0508 (4)	0.40508 (9)	0.0198 (7)	
C2	0.57813 (13)	0.9369 (4)	0.41662 (10)	0.0183 (7)	
C3	0.62505 (13)	0.9098 (4)	0.38905 (9)	0.0184 (7)	
C4	0.62789 (13)	1.0431 (5)	0.35943 (10)	0.0223 (7)	
H4A	0.6348	1.1388	0.3741	0.027*	
H4B	0.6565	1.0265	0.3399	0.027*	
C5	0.54139 (13)	1.1343 (4)	0.36490 (10)	0.0211 (7)	
H5A	0.5071	1.1448	0.3513	0.025*	
H5B	0.5551	1.2376	0.3702	0.025*	
C6	0.48014 (13)	0.9751 (4)	0.41316 (9)	0.0192 (7)	
H6	0.4852	0.8633	0.4166	0.023*	
C7	0.46662 (13)	1.0452 (4)	0.45370 (10)	0.0196 (7)	
C8	0.41806 (13)	1.0165 (4)	0.47611 (10)	0.0223 (7)	
C9	0.38395 (15)	1.1351 (4)	0.48791 (11)	0.0254 (8)	
C10	0.33538 (15)	1.1067 (5)	0.50485 (11)	0.0283 (8)	
H10	0.3126	1.1885	0.5112	0.034*	
C11	0.32043 (15)	0.9595 (5)	0.51246 (11)	0.0304 (9)	
H11	0.2874	0.9404	0.5245	0.036*	
C12	0.35266 (15)	0.8391 (5)	0.50285 (10)	0.0262 (8)	
H12	0.3424	0.7378	0.5088	0.031*	
C13	0.40018 (14)	0.8682 (4)	0.48447 (10)	0.0224 (7)	
C14	0.43769 (13)	1.0022 (4)	0.38233 (9)	0.0193 (7)	
C15	0.42652 (14)	0.8877 (4)	0.35449 (11)	0.0249 (8)	
H15	0.4436	0.7922	0.3564	0.030*	
C16	0.39001 (17)	0.9140 (5)	0.32358 (12)	0.0356 (10)	
H16	0.3827	0.8374	0.3042	0.043*	
C17	0.36537 (17)	1.0505 (5)	0.32185 (13)	0.0370 (10)	
C18	0.37413 (15)	1.1647 (5)	0.35003 (13)	0.0346 (9)	
H18	0.3557	1.2583	0.3486	0.042*	
C19	0.41041 (14)	1.1385 (4)	0.38032 (11)	0.0251 (8)	
H19	0.4167	1.2148	0.4000	0.030*	
C20	0.58312 (14)	1.1354 (5)	0.29984 (10)	0.0261 (8)	
H20A	0.6137	1.0941	0.2852	0.031*	
H20B	0.5904	1.2435	0.3058	0.031*	
C21	0.53567 (14)	1.1266 (4)	0.27248 (10)	0.0239 (8)	
C22	0.50555 (15)	0.9968 (5)	0.26976 (11)	0.0297 (9)	
H22	0.5118	0.9144	0.2876	0.036*	
C23	0.46609 (17)	0.9858 (5)	0.24113 (13)	0.0370 (10)	
H23	0.4456	0.8961	0.2395	0.044*	
C24	0.45640 (17)	1.1063 (6)	0.21469 (12)	0.0379 (10)	
H24	0.4298	1.0982	0.1948	0.046*	
C25	0.48567 (16)	1.2362 (5)	0.21780 (11)	0.0331 (9)	
H25	0.4791	1.3189	0.2001	0.040*	
C26	0.52473 (14)	1.2475 (5)	0.24656 (11)	0.0282 (8)	
H26	0.5443	1.3388	0.2487	0.034*	
C27	0.61786 (13)	0.7504 (4)	0.36647 (10)	0.0203 (7)	
C28	0.70261 (14)	0.7521 (4)	0.39313 (11)	0.0264 (8)	
H28A	0.7203	0.7836	0.3680	0.032*	
H28B	0.7283	0.7019	0.4111	0.032*	
C29	0.67655 (13)	0.8865 (4)	0.41392 (10)	0.0221 (7)	
H29	0.6659	0.8514	0.4414	0.027*	
C30	0.70972 (13)	1.0271 (5)	0.41960 (10)	0.0241 (8)	
C31	0.75910 (14)	1.0404 (5)	0.40336 (12)	0.0326 (9)	
H31	0.7733	0.9583	0.3883	0.039*	
C32	0.78855 (15)	1.1720 (6)	0.40857 (13)	0.0400 (10)	
H32	0.8224	1.1797	0.3969	0.048*	
C33	0.76842 (16)	1.2909 (5)	0.43067 (12)	0.0339 (9)	
C34	0.71960 (16)	1.2802 (5)	0.44791 (12)	0.0334 (9)	
H34	0.7058	1.3623	0.4631	0.040*	
C35	0.69082 (15)	1.1476 (5)	0.44278 (11)	0.0303 (9)	
H35	0.6576	1.1388	0.4553	0.036*	
C36	0.67124 (17)	0.5140 (5)	0.36293 (13)	0.0339 (9)	
H36A	0.6847	0.5401	0.3362	0.051*	
H36B	0.6398	0.4513	0.3601	0.051*	
H36C	0.6979	0.4573	0.3779	0.051*	
C37	0.62585 (14)	0.7643 (4)	0.31973 (10)	0.0242 (8)	
C38	0.54876 (14)	0.6387 (4)	0.32859 (10)	0.0231 (7)	
C39	0.56571 (14)	0.6681 (4)	0.36807 (10)	0.0217 (7)	
C40	0.53884 (15)	0.6023 (4)	0.39984 (11)	0.0261 (8)	
H40	0.5507	0.6152	0.4267	0.031*	
C41	0.49401 (15)	0.5165 (4)	0.39183 (12)	0.0287 (8)	
H41	0.4750	0.4727	0.4135	0.034*	
C42	0.47716 (15)	0.4949 (4)	0.35261 (12)	0.0276 (8)	
H42	0.4462	0.4382	0.3477	0.033*	
C43	0.50477 (15)	0.5549 (4)	0.32021 (11)	0.0277 (8)	
H43	0.4936	0.5385	0.2933	0.033*	
C44	0.79140 (13)	0.4535 (4)	0.61487 (10)	0.0193 (7)	
C45	0.83470 (14)	0.5699 (4)	0.60303 (10)	0.0201 (7)	
C46	0.88109 (13)	0.6011 (4)	0.63086 (10)	0.0198 (7)	
C47	0.88525 (13)	0.4686 (4)	0.66056 (10)	0.0224 (7)	
H47A	0.8929	0.3732	0.6459	0.027*	
H47B	0.9139	0.4874	0.6800	0.027*	
C48	0.79913 (14)	0.3729 (4)	0.65574 (10)	0.0228 (7)	
H48A	0.7649	0.3634	0.6694	0.027*	
H48B	0.8128	0.2692	0.6510	0.027*	
C49	0.73631 (13)	0.5233 (4)	0.60600 (9)	0.0190 (7)	
H49	0.7405	0.6344	0.6006	0.023*	
C50	0.72359 (13)	0.4432 (4)	0.56665 (10)	0.0193 (7)	
C51	0.67364 (14)	0.4640 (4)	0.54461 (10)	0.0229 (7)	
C52	0.63953 (15)	0.3428 (5)	0.53677 (11)	0.0271 (8)	
C53	0.59018 (16)	0.3613 (5)	0.51950 (11)	0.0339 (10)	
H53	0.5682	0.2765	0.5144	0.041*	
C54	0.57427 (15)	0.5080 (6)	0.50999 (12)	0.0400 (11)	
H54	0.5400	0.5243	0.4998	0.048*	
C55	0.60715 (16)	0.6298 (5)	0.51511 (11)	0.0318 (9)	
H55	0.5966	0.7288	0.5072	0.038*	
C56	0.65580 (15)	0.6062 (4)	0.53197 (10)	0.0264 (8)	
C57	0.69461 (13)	0.5022 (4)	0.63777 (10)	0.0196 (7)	
C58	0.68451 (15)	0.6202 (5)	0.66496 (11)	0.0277 (8)	
H58	0.7014	0.7154	0.6616	0.033*	
C59	0.65006 (16)	0.5992 (5)	0.69671 (12)	0.0314 (9)	
H59	0.6436	0.6789	0.7154	0.038*	
C60	0.62538 (16)	0.4612 (6)	0.70081 (12)	0.0354 (10)	
C61	0.63297 (15)	0.3444 (5)	0.67353 (12)	0.0304 (9)	
H61	0.6145	0.2513	0.6762	0.037*	
C62	0.66825 (14)	0.3660 (4)	0.64202 (11)	0.0263 (8)	
H62	0.6742	0.2864	0.6233	0.032*	
C63	0.84205 (14)	0.3750 (5)	0.72034 (10)	0.0242 (7)	
H63A	0.8716	0.4215	0.7351	0.029*	
H63B	0.8515	0.2682	0.7145	0.029*	
C64	0.79431 (13)	0.3761 (4)	0.74757 (10)	0.0229 (7)	
C65	0.76286 (15)	0.5042 (5)	0.75104 (11)	0.0298 (8)	
H65	0.7689	0.5892	0.7340	0.036*	
C66	0.72235 (16)	0.5091 (6)	0.77933 (13)	0.0383 (10)	
H66	0.7008	0.5967	0.7813	0.046*	
C67	0.71373 (16)	0.3854 (6)	0.80454 (12)	0.0374 (10)	
H67	0.6865	0.3884	0.8240	0.045*	
C68	0.74494 (16)	0.2583 (5)	0.80107 (12)	0.0356 (10)	
H68	0.7394	0.1740	0.8184	0.043*	
C69	0.78424 (15)	0.2527 (5)	0.77249 (10)	0.0261 (8)	
H69	0.8047	0.1631	0.7699	0.031*	
C70	0.87270 (14)	0.7620 (4)	0.65290 (10)	0.0211 (7)	
C71	0.95743 (14)	0.7652 (5)	0.62621 (11)	0.0262 (8)	
H71A	0.9755	0.7355	0.6514	0.031*	
H71B	0.9825	0.8169	0.6081	0.031*	
C72	0.93233 (13)	0.6284 (4)	0.60569 (10)	0.0217 (7)	
H72	0.9213	0.6618	0.5782	0.026*	
C73	0.96695 (14)	0.4906 (4)	0.60042 (10)	0.0236 (8)	
C74	1.01649 (15)	0.4805 (5)	0.61742 (11)	0.0319 (9)	
H74	1.0296	0.5642	0.6324	0.038*	
C75	1.04736 (16)	0.3514 (5)	0.61315 (12)	0.0348 (10)	
H75	1.0811	0.3470	0.6251	0.042*	
C76	1.02857 (16)	0.2313 (5)	0.59157 (12)	0.0358 (10)	
C77	0.97972 (16)	0.2368 (5)	0.57354 (12)	0.0350 (9)	
H77	0.9670	0.1531	0.5584	0.042*	
C78	0.94980 (15)	0.3666 (5)	0.57807 (11)	0.0307 (9)	
H78	0.9165	0.3712	0.5655	0.037*	
C79	0.92313 (16)	1.0007 (5)	0.65661 (12)	0.0335 (9)	
H79A	0.9371	0.9756	0.6833	0.050*	
H79B	0.8907	1.0590	0.6596	0.050*	
H79C	0.9488	1.0612	0.6417	0.050*	
C80	0.88097 (14)	0.7476 (4)	0.69968 (10)	0.0224 (7)	
C81	0.80291 (15)	0.8700 (4)	0.69060 (10)	0.0242 (8)	
C82	0.81982 (14)	0.8396 (4)	0.65138 (10)	0.0205 (7)	
C83	0.79248 (15)	0.9015 (4)	0.61928 (10)	0.0253 (8)	
H83	0.8045	0.8873	0.5925	0.030*	
C84	0.74746 (16)	0.9845 (5)	0.62673 (11)	0.0295 (8)	
H84	0.7282	1.0256	0.6048	0.035*	
C85	0.73009 (16)	1.0081 (5)	0.66622 (12)	0.0303 (8)	
H85	0.6986	1.0623	0.6708	0.036*	
C86	0.75858 (15)	0.9528 (4)	0.69889 (11)	0.0268 (8)	
H86	0.7478	0.9716	0.7258	0.032*	

### Atomic displacement parameters (Å^2^)

**Table:**

	*U*^11^	*U*^22^	*U*^33^	*U*^12^	*U*^13^	*U*^23^
Cl1	0.0311 (5)	0.0255 (5)	0.0383 (5)	0.0028 (4)	0.0114 (4)	−0.0008 (4)
Cl2	0.0335 (5)	0.0263 (5)	0.0339 (4)	0.0032 (4)	0.0095 (4)	0.0046 (4)
Cl3	0.0757 (9)	0.0570 (8)	0.0770 (9)	−0.0234 (7)	−0.0587 (8)	0.0254 (7)
Cl4	0.0384 (6)	0.0441 (7)	0.0687 (8)	−0.0124 (5)	−0.0201 (5)	0.0121 (6)
Cl5	0.0363 (5)	0.0324 (5)	0.0383 (5)	−0.0095 (4)	−0.0095 (4)	0.0021 (4)
Cl6	0.0390 (5)	0.0309 (5)	0.0375 (5)	−0.0006 (4)	−0.0046 (4)	0.0103 (4)
Cl7	0.0486 (6)	0.0523 (7)	0.0513 (6)	0.0114 (5)	0.0312 (5)	0.0121 (5)
Cl8	0.0383 (6)	0.0486 (7)	0.0673 (7)	0.0163 (5)	0.0163 (5)	0.0060 (6)
N1	0.0177 (14)	0.0324 (17)	0.0188 (13)	0.0003 (13)	0.0047 (11)	−0.0002 (12)
N2	0.0122 (13)	0.0309 (17)	0.0153 (13)	−0.0001 (12)	−0.0013 (10)	0.0037 (11)
N3	0.0230 (15)	0.0328 (18)	0.0223 (15)	0.0080 (14)	−0.0031 (12)	0.0004 (12)
N4	0.0259 (15)	0.0313 (18)	0.0152 (14)	0.0008 (14)	−0.0036 (11)	−0.0002 (12)
N5	0.0216 (15)	0.0264 (16)	0.0186 (13)	−0.0011 (13)	−0.0039 (11)	−0.0037 (11)
N6	0.0172 (14)	0.0323 (18)	0.0173 (13)	0.0018 (13)	−0.0014 (11)	0.0022 (12)
N7	0.0236 (15)	0.0285 (17)	0.0220 (14)	−0.0067 (13)	0.0038 (12)	−0.0042 (12)
N8	0.0245 (15)	0.0361 (19)	0.0149 (13)	0.0017 (14)	0.0006 (12)	−0.0023 (13)
O1	0.0162 (11)	0.0264 (13)	0.0221 (11)	−0.0071 (10)	0.0067 (9)	−0.0041 (10)
O2	0.0234 (13)	0.0345 (15)	0.0166 (11)	0.0050 (11)	0.0012 (9)	0.0066 (10)
O3	0.0226 (13)	0.0400 (16)	0.0185 (12)	−0.0018 (12)	0.0023 (9)	−0.0023 (11)
O4	0.0195 (12)	0.0253 (14)	0.0242 (12)	0.0022 (10)	−0.0051 (10)	−0.0067 (10)
O5	0.0222 (12)	0.0373 (15)	0.0192 (12)	−0.0010 (11)	−0.0009 (10)	0.0030 (11)
O6	0.0223 (13)	0.0407 (16)	0.0184 (11)	−0.0014 (12)	−0.0015 (10)	−0.0009 (10)
C1	0.0183 (16)	0.0254 (19)	0.0156 (15)	0.0014 (14)	0.0004 (12)	0.0006 (13)
C2	0.0154 (16)	0.0220 (18)	0.0175 (15)	−0.0007 (14)	−0.0026 (12)	0.0004 (13)
C3	0.0144 (15)	0.0279 (19)	0.0128 (14)	0.0038 (14)	−0.0018 (12)	0.0007 (13)
C4	0.0160 (17)	0.035 (2)	0.0157 (15)	−0.0017 (15)	−0.0001 (12)	0.0025 (14)
C5	0.0160 (16)	0.027 (2)	0.0200 (16)	0.0026 (14)	0.0024 (12)	0.0045 (14)
C6	0.0198 (16)	0.0199 (18)	0.0180 (15)	0.0036 (14)	0.0023 (13)	0.0004 (13)
C7	0.0180 (16)	0.0242 (18)	0.0165 (15)	0.0028 (14)	0.0019 (12)	0.0002 (12)
C8	0.0154 (15)	0.036 (2)	0.0154 (15)	−0.0058 (15)	−0.0007 (12)	−0.0012 (14)
C9	0.0267 (19)	0.029 (2)	0.0208 (16)	−0.0013 (16)	0.0011 (14)	−0.0015 (14)
C10	0.0215 (18)	0.039 (2)	0.0244 (17)	−0.0001 (17)	0.0045 (14)	−0.0091 (16)
C11	0.0221 (18)	0.046 (2)	0.0231 (18)	−0.0059 (18)	0.0088 (14)	−0.0052 (16)
C12	0.0288 (19)	0.032 (2)	0.0178 (16)	−0.0109 (16)	0.0031 (14)	−0.0037 (14)
C13	0.0229 (18)	0.027 (2)	0.0176 (15)	0.0015 (15)	−0.0004 (13)	−0.0026 (13)
C14	0.0164 (15)	0.0255 (19)	0.0161 (15)	−0.0011 (15)	0.0011 (12)	0.0008 (13)
C15	0.0236 (18)	0.026 (2)	0.0250 (17)	−0.0054 (16)	−0.0018 (14)	0.0002 (15)
C16	0.036 (2)	0.042 (3)	0.029 (2)	−0.016 (2)	−0.0129 (17)	−0.0025 (18)
C17	0.035 (2)	0.036 (2)	0.039 (2)	−0.0099 (19)	−0.0204 (18)	0.0119 (18)
C18	0.0225 (19)	0.036 (2)	0.046 (2)	−0.0018 (17)	−0.0060 (17)	0.0115 (19)
C19	0.0210 (17)	0.028 (2)	0.0267 (17)	−0.0033 (16)	−0.0006 (14)	0.0013 (14)
C20	0.0227 (17)	0.037 (2)	0.0186 (16)	−0.0016 (17)	0.0018 (14)	0.0080 (15)
C21	0.0209 (17)	0.035 (2)	0.0162 (15)	0.0048 (16)	0.0026 (13)	0.0037 (14)
C22	0.0254 (19)	0.036 (2)	0.0282 (18)	−0.0026 (18)	−0.0064 (15)	0.0049 (16)
C23	0.035 (2)	0.040 (3)	0.036 (2)	−0.003 (2)	−0.0102 (18)	0.0049 (18)
C24	0.029 (2)	0.057 (3)	0.0271 (18)	0.005 (2)	−0.0114 (16)	0.0000 (18)
C25	0.029 (2)	0.048 (3)	0.0227 (17)	0.0109 (19)	−0.0007 (15)	0.0078 (17)
C26	0.0230 (18)	0.037 (2)	0.0249 (17)	0.0060 (16)	0.0026 (15)	0.0083 (16)
C27	0.0178 (16)	0.027 (2)	0.0159 (15)	0.0016 (14)	0.0007 (12)	−0.0011 (13)
C28	0.0205 (17)	0.037 (2)	0.0214 (16)	0.0066 (16)	−0.0031 (13)	0.0008 (15)
C29	0.0157 (16)	0.035 (2)	0.0158 (15)	0.0065 (15)	−0.0026 (12)	0.0012 (14)
C30	0.0190 (17)	0.040 (2)	0.0136 (15)	−0.0015 (16)	−0.0068 (12)	0.0006 (14)
C31	0.0198 (18)	0.048 (3)	0.0302 (19)	−0.0002 (18)	−0.0001 (15)	0.0022 (18)
C32	0.0192 (19)	0.063 (3)	0.038 (2)	−0.003 (2)	−0.0019 (16)	0.006 (2)
C33	0.033 (2)	0.038 (2)	0.0314 (19)	−0.0047 (18)	−0.0120 (17)	0.0037 (17)
C34	0.029 (2)	0.039 (2)	0.0327 (19)	−0.0004 (18)	−0.0050 (16)	−0.0026 (17)
C35	0.0239 (18)	0.042 (2)	0.0247 (17)	−0.0024 (17)	−0.0009 (15)	−0.0066 (16)
C36	0.037 (2)	0.032 (2)	0.033 (2)	0.0097 (18)	−0.0045 (17)	−0.0062 (17)
C37	0.0220 (17)	0.031 (2)	0.0191 (16)	0.0053 (16)	0.0009 (13)	−0.0031 (14)
C38	0.0218 (17)	0.029 (2)	0.0189 (15)	0.0064 (15)	−0.0015 (13)	−0.0012 (13)
C39	0.0200 (17)	0.0264 (19)	0.0188 (16)	0.0042 (15)	−0.0013 (13)	−0.0009 (13)
C40	0.031 (2)	0.023 (2)	0.0244 (17)	0.0002 (16)	0.0031 (15)	0.0008 (14)
C41	0.033 (2)	0.0201 (19)	0.0330 (19)	−0.0019 (17)	0.0091 (16)	0.0033 (15)
C42	0.0247 (18)	0.0208 (19)	0.037 (2)	0.0021 (16)	−0.0025 (16)	−0.0029 (16)
C43	0.0286 (19)	0.029 (2)	0.0259 (17)	0.0031 (17)	−0.0050 (15)	−0.0051 (15)
C44	0.0171 (16)	0.0237 (19)	0.0170 (15)	0.0033 (14)	−0.0015 (12)	−0.0037 (13)
C45	0.0202 (17)	0.0240 (19)	0.0159 (15)	0.0000 (14)	0.0020 (12)	−0.0014 (13)
C46	0.0167 (16)	0.0247 (19)	0.0178 (15)	0.0011 (14)	0.0006 (12)	0.0019 (13)
C47	0.0135 (16)	0.034 (2)	0.0197 (16)	−0.0021 (14)	0.0017 (12)	0.0010 (14)
C48	0.0177 (16)	0.028 (2)	0.0232 (16)	0.0009 (15)	−0.0044 (13)	0.0039 (14)
C49	0.0181 (16)	0.0252 (19)	0.0137 (14)	0.0054 (14)	0.0000 (12)	−0.0016 (13)
C50	0.0146 (15)	0.0266 (19)	0.0167 (14)	0.0004 (14)	0.0022 (12)	0.0029 (13)
C51	0.0211 (17)	0.031 (2)	0.0165 (15)	−0.0021 (15)	−0.0001 (13)	0.0023 (13)
C52	0.0214 (18)	0.039 (2)	0.0206 (16)	−0.0067 (17)	−0.0070 (14)	0.0004 (15)
C53	0.026 (2)	0.052 (3)	0.0238 (19)	−0.0073 (19)	−0.0087 (15)	0.0017 (17)
C54	0.022 (2)	0.073 (3)	0.025 (2)	0.007 (2)	−0.0047 (15)	0.010 (2)
C55	0.031 (2)	0.043 (2)	0.0209 (17)	0.0121 (19)	−0.0033 (15)	0.0104 (16)
C56	0.030 (2)	0.032 (2)	0.0170 (15)	0.0005 (17)	0.0013 (14)	0.0017 (15)
C57	0.0173 (16)	0.0251 (19)	0.0166 (14)	0.0019 (14)	0.0003 (12)	0.0053 (13)
C58	0.0234 (18)	0.030 (2)	0.0298 (18)	0.0012 (16)	0.0015 (15)	0.0001 (15)
C59	0.030 (2)	0.037 (2)	0.0279 (19)	0.0077 (18)	0.0057 (16)	−0.0012 (16)
C60	0.025 (2)	0.046 (3)	0.035 (2)	0.0063 (19)	0.0117 (17)	0.0111 (18)
C61	0.0231 (18)	0.029 (2)	0.040 (2)	0.0047 (16)	0.0063 (16)	0.0135 (16)
C62	0.0210 (18)	0.029 (2)	0.0286 (18)	0.0024 (15)	0.0017 (14)	0.0011 (15)
C63	0.0186 (16)	0.034 (2)	0.0202 (16)	0.0032 (16)	0.0011 (13)	0.0059 (15)
C64	0.0190 (16)	0.032 (2)	0.0178 (15)	−0.0026 (15)	−0.0016 (13)	0.0022 (14)
C65	0.0280 (19)	0.034 (2)	0.0270 (19)	0.0040 (18)	0.0046 (15)	0.0083 (16)
C66	0.031 (2)	0.042 (3)	0.041 (2)	0.006 (2)	0.0082 (18)	−0.0003 (19)
C67	0.028 (2)	0.057 (3)	0.0266 (19)	−0.007 (2)	0.0083 (16)	0.0031 (19)
C68	0.031 (2)	0.051 (3)	0.0247 (18)	−0.010 (2)	0.0022 (16)	0.0116 (18)
C69	0.0245 (18)	0.032 (2)	0.0222 (16)	0.0009 (16)	−0.0017 (13)	0.0045 (14)
C70	0.0212 (17)	0.026 (2)	0.0163 (15)	−0.0049 (15)	0.0016 (13)	−0.0016 (13)
C71	0.0172 (17)	0.039 (2)	0.0230 (16)	−0.0067 (16)	0.0022 (13)	−0.0052 (15)
C72	0.0174 (16)	0.033 (2)	0.0145 (15)	−0.0008 (15)	−0.0004 (12)	−0.0017 (13)
C73	0.0187 (17)	0.033 (2)	0.0194 (16)	−0.0012 (16)	0.0027 (13)	−0.0001 (14)
C74	0.0215 (18)	0.048 (3)	0.0263 (18)	−0.0052 (19)	−0.0021 (15)	0.0012 (17)
C75	0.0209 (19)	0.050 (3)	0.034 (2)	0.0040 (18)	−0.0005 (16)	0.0011 (18)
C76	0.030 (2)	0.040 (3)	0.037 (2)	0.0104 (19)	0.0123 (17)	0.0080 (18)
C77	0.031 (2)	0.040 (2)	0.034 (2)	0.0011 (19)	0.0073 (17)	−0.0073 (17)
C78	0.0239 (18)	0.043 (2)	0.0248 (18)	0.0043 (17)	0.0000 (15)	−0.0027 (16)
C79	0.040 (2)	0.033 (2)	0.027 (2)	−0.0118 (19)	0.0028 (17)	−0.0044 (16)
C80	0.0229 (17)	0.026 (2)	0.0185 (15)	−0.0044 (15)	0.0000 (13)	−0.0010 (13)
C81	0.0262 (18)	0.0244 (19)	0.0219 (16)	−0.0047 (16)	−0.0007 (14)	−0.0022 (14)
C82	0.0255 (17)	0.0189 (18)	0.0172 (15)	−0.0038 (15)	0.0038 (13)	0.0000 (12)
C83	0.0305 (19)	0.026 (2)	0.0189 (16)	−0.0002 (16)	−0.0004 (14)	0.0023 (14)
C84	0.031 (2)	0.032 (2)	0.0259 (18)	0.0022 (18)	−0.0022 (15)	0.0019 (15)
C85	0.030 (2)	0.025 (2)	0.036 (2)	0.0067 (17)	0.0040 (16)	0.0011 (16)
C86	0.029 (2)	0.026 (2)	0.0257 (17)	−0.0023 (16)	0.0055 (15)	−0.0010 (15)

### Geometric parameters (Å, °)

**Table:**

Cl1—C9	1.737 (4)	C32—H32	0.9500
Cl2—C13	1.739 (4)	C33—C34	1.377 (6)
Cl3—C17	1.742 (4)	C34—C35	1.390 (6)
Cl4—C33	1.740 (4)	C34—H34	0.9500
Cl5—C52	1.749 (4)	C35—H35	0.9500
Cl6—C56	1.752 (4)	C36—H36A	0.9800
Cl7—C60	1.744 (4)	C36—H36B	0.9800
Cl8—C76	1.742 (4)	C36—H36C	0.9800
N1—C7	1.289 (5)	C38—C43	1.374 (5)
N1—O1	1.411 (3)	C38—C39	1.404 (5)
N2—C4	1.467 (4)	C39—C40	1.386 (5)
N2—C5	1.469 (4)	C40—C41	1.399 (5)
N2—C20	1.474 (4)	C40—H40	0.9500
N3—C28	1.455 (5)	C41—C42	1.384 (6)
N3—C36	1.464 (5)	C41—H41	0.9500
N3—C27	1.474 (4)	C42—C43	1.391 (5)
N4—C37	1.354 (5)	C42—H42	0.9500
N4—C38	1.396 (5)	C43—H43	0.9500
N4—H4	0.87 (4)	C44—C48	1.544 (5)
N5—C50	1.272 (4)	C44—C45	1.560 (5)
N5—O4	1.396 (4)	C44—C49	1.566 (4)
N6—C48	1.465 (4)	C45—C46	1.529 (5)
N6—C47	1.465 (4)	C46—C47	1.531 (5)
N6—C63	1.469 (4)	C46—C72	1.573 (5)
N7—C71	1.451 (5)	C46—C70	1.608 (5)
N7—C70	1.466 (4)	C47—H47A	0.9900
N7—C79	1.467 (5)	C47—H47B	0.9900
N8—C80	1.350 (5)	C48—H48A	0.9900
N8—C81	1.404 (5)	C48—H48B	0.9900
N8—H8	0.68 (5)	C49—C57	1.512 (4)
O1—C1	1.474 (4)	C49—C50	1.520 (4)
O2—C2	1.211 (4)	C49—H49	1.0000
O3—C37	1.227 (4)	C50—C51	1.484 (5)
O4—C44	1.471 (4)	C51—C56	1.396 (5)
O5—C45	1.213 (4)	C51—C52	1.403 (5)
O6—C80	1.224 (4)	C52—C53	1.396 (5)
C1—C5	1.535 (4)	C53—C54	1.390 (7)
C1—C6	1.552 (5)	C53—H53	0.9500
C1—C2	1.560 (5)	C54—C55	1.373 (7)
C2—C3	1.528 (4)	C54—H54	0.9500
C3—C4	1.532 (5)	C55—C56	1.381 (5)
C3—C29	1.569 (4)	C55—H55	0.9500
C3—C27	1.601 (5)	C57—C62	1.383 (5)
C4—H4A	0.9900	C57—C58	1.400 (5)
C4—H4B	0.9900	C58—C59	1.386 (5)
C5—H5A	0.9900	C58—H58	0.9500
C5—H5B	0.9900	C59—C60	1.376 (6)
C6—C14	1.511 (4)	C59—H59	0.9500
C6—C7	1.520 (4)	C60—C61	1.384 (6)
C6—H6	1.0000	C61—C62	1.395 (5)
C7—C8	1.470 (5)	C61—H61	0.9500
C8—C13	1.411 (5)	C62—H62	0.9500
C8—C9	1.416 (5)	C63—C64	1.520 (5)
C9—C10	1.387 (5)	C63—H63A	0.9900
C10—C11	1.374 (6)	C63—H63B	0.9900
C10—H10	0.9500	C64—C69	1.390 (5)
C11—C12	1.381 (6)	C64—C65	1.390 (5)
C11—H11	0.9500	C65—C66	1.399 (5)
C12—C13	1.384 (5)	C65—H65	0.9500
C12—H12	0.9500	C66—C67	1.391 (6)
C14—C19	1.389 (5)	C66—H66	0.9500
C14—C15	1.397 (5)	C67—C68	1.380 (6)
C15—C16	1.407 (5)	C67—H67	0.9500
C15—H15	0.9500	C68—C69	1.383 (5)
C16—C17	1.358 (6)	C68—H68	0.9500
C16—H16	0.9500	C69—H69	0.9500
C17—C18	1.392 (6)	C70—C82	1.517 (5)
C18—C19	1.388 (5)	C70—C80	1.572 (4)
C18—H18	0.9500	C71—C72	1.525 (5)
C19—H19	0.9500	C71—H71A	0.9900
C20—C21	1.518 (5)	C71—H71B	0.9900
C20—H20A	0.9900	C72—C73	1.513 (5)
C20—H20B	0.9900	C72—H72	1.0000
C21—C22	1.381 (6)	C73—C78	1.390 (5)
C21—C26	1.397 (5)	C73—C74	1.391 (5)
C22—C23	1.390 (5)	C74—C75	1.391 (6)
C22—H22	0.9500	C74—H74	0.9500
C23—C24	1.398 (6)	C75—C76	1.365 (6)
C23—H23	0.9500	C75—H75	0.9500
C24—C25	1.371 (6)	C76—C77	1.387 (6)
C24—H24	0.9500	C77—C78	1.383 (6)
C25—C26	1.386 (5)	C77—H77	0.9500
C25—H25	0.9500	C78—H78	0.9500
C26—H26	0.9500	C79—H79A	0.9800
C27—C39	1.520 (5)	C79—H79B	0.9800
C27—C37	1.570 (4)	C79—H79C	0.9800
C28—C29	1.523 (5)	C81—C86	1.376 (5)
C28—H28A	0.9900	C81—C82	1.398 (5)
C28—H28B	0.9900	C82—C83	1.386 (5)
C29—C30	1.513 (5)	C83—C84	1.386 (5)
C29—H29	1.0000	C83—H83	0.9500
C30—C31	1.379 (5)	C84—C85	1.400 (5)
C30—C35	1.397 (5)	C84—H84	0.9500
C31—C32	1.393 (6)	C85—C86	1.394 (5)
C31—H31	0.9500	C85—H85	0.9500
C32—C33	1.378 (6)	C86—H86	0.9500
			
C7—N1—O1	109.3 (3)	C39—C40—H40	120.4
C4—N2—C5	107.1 (3)	C41—C40—H40	120.4
C4—N2—C20	111.1 (3)	C42—C41—C40	120.5 (4)
C5—N2—C20	109.9 (3)	C42—C41—H41	119.7
C28—N3—C36	114.7 (3)	C40—C41—H41	119.7
C28—N3—C27	106.2 (3)	C41—C42—C43	121.1 (4)
C36—N3—C27	116.1 (3)	C41—C42—H42	119.4
C37—N4—C38	112.0 (3)	C43—C42—H42	119.4
C37—N4—H4	122 (3)	C38—C43—C42	117.6 (3)
C38—N4—H4	126 (3)	C38—C43—H43	121.2
C50—N5—O4	110.2 (3)	C42—C43—H43	121.2
C48—N6—C47	107.6 (3)	O4—C44—C48	105.8 (3)
C48—N6—C63	110.4 (3)	O4—C44—C45	102.8 (3)
C47—N6—C63	110.9 (3)	C48—C44—C45	115.6 (3)
C71—N7—C70	106.9 (3)	O4—C44—C49	103.8 (2)
C71—N7—C79	115.4 (3)	C48—C44—C49	117.4 (3)
C70—N7—C79	114.7 (3)	C45—C44—C49	109.5 (3)
C80—N8—C81	112.5 (3)	O5—C45—C46	122.2 (3)
C80—N8—H8	124 (4)	O5—C45—C44	116.6 (3)
C81—N8—H8	123 (4)	C46—C45—C44	121.2 (3)
N1—O1—C1	110.2 (2)	C45—C46—C47	107.8 (3)
N5—O4—C44	110.5 (2)	C45—C46—C72	110.7 (3)
O1—C1—C5	105.8 (3)	C47—C46—C72	113.6 (3)
O1—C1—C6	104.2 (2)	C45—C46—C70	109.2 (3)
C5—C1—C6	117.8 (3)	C47—C46—C70	112.8 (3)
O1—C1—C2	102.7 (2)	C72—C46—C70	102.6 (3)
C5—C1—C2	115.5 (3)	N6—C47—C46	108.1 (3)
C6—C1—C2	109.0 (3)	N6—C47—H47A	110.1
O2—C2—C3	121.8 (3)	C46—C47—H47A	110.1
O2—C2—C1	116.6 (3)	N6—C47—H47B	110.1
C3—C2—C1	121.5 (3)	C46—C47—H47B	110.1
C2—C3—C4	107.6 (3)	H47A—C47—H47B	108.4
C2—C3—C29	111.4 (3)	N6—C48—C44	112.0 (3)
C4—C3—C29	113.5 (3)	N6—C48—H48A	109.2
C2—C3—C27	109.1 (3)	C44—C48—H48A	109.2
C4—C3—C27	112.1 (3)	N6—C48—H48B	109.2
C29—C3—C27	103.2 (3)	C44—C48—H48B	109.2
N2—C4—C3	108.1 (3)	H48A—C48—H48B	107.9
N2—C4—H4A	110.1	C57—C49—C50	113.1 (3)
C3—C4—H4A	110.1	C57—C49—C44	117.1 (3)
N2—C4—H4B	110.1	C50—C49—C44	99.9 (3)
C3—C4—H4B	110.1	C57—C49—H49	108.7
H4A—C4—H4B	108.4	C50—C49—H49	108.7
N2—C5—C1	111.6 (3)	C44—C49—H49	108.7
N2—C5—H5A	109.3	N5—C50—C51	121.4 (3)
C1—C5—H5A	109.3	N5—C50—C49	114.7 (3)
N2—C5—H5B	109.3	C51—C50—C49	123.4 (3)
C1—C5—H5B	109.3	C56—C51—C52	115.0 (3)
H5A—C5—H5B	108.0	C56—C51—C50	122.7 (3)
C14—C6—C7	111.8 (3)	C52—C51—C50	122.2 (3)
C14—C6—C1	116.9 (3)	C53—C52—C51	123.3 (4)
C7—C6—C1	100.4 (3)	C53—C52—Cl5	115.5 (3)
C14—C6—H6	109.1	C51—C52—Cl5	121.2 (3)
C7—C6—H6	109.1	C54—C53—C52	117.8 (4)
C1—C6—H6	109.1	C54—C53—H53	121.1
N1—C7—C8	120.3 (3)	C52—C53—H53	121.1
N1—C7—C6	114.5 (3)	C55—C54—C53	121.2 (4)
C8—C7—C6	124.8 (3)	C55—C54—H54	119.4
C13—C8—C9	115.3 (3)	C53—C54—H54	119.4
C13—C8—C7	122.2 (3)	C54—C55—C56	119.0 (4)
C9—C8—C7	122.3 (3)	C54—C55—H55	120.5
C10—C9—C8	122.1 (4)	C56—C55—H55	120.5
C10—C9—Cl1	116.4 (3)	C55—C56—C51	123.5 (4)
C8—C9—Cl1	121.4 (3)	C55—C56—Cl6	117.5 (3)
C11—C10—C9	119.6 (4)	C51—C56—Cl6	119.1 (3)
C11—C10—H10	120.2	C62—C57—C58	119.2 (3)
C9—C10—H10	120.2	C62—C57—C49	121.4 (3)
C10—C11—C12	121.0 (4)	C58—C57—C49	119.3 (3)
C10—C11—H11	119.5	C59—C58—C57	120.6 (4)
C12—C11—H11	119.5	C59—C58—H58	119.7
C11—C12—C13	119.0 (4)	C57—C58—H58	119.7
C11—C12—H12	120.5	C60—C59—C58	119.0 (4)
C13—C12—H12	120.5	C60—C59—H59	120.5
C12—C13—C8	122.9 (3)	C58—C59—H59	120.5
C12—C13—Cl2	117.2 (3)	C59—C60—C61	121.8 (4)
C8—C13—Cl2	119.9 (3)	C59—C60—Cl7	119.2 (3)
C19—C14—C15	119.2 (3)	C61—C60—Cl7	119.0 (3)
C19—C14—C6	122.0 (3)	C60—C61—C62	118.7 (4)
C15—C14—C6	118.8 (3)	C60—C61—H61	120.7
C14—C15—C16	120.0 (4)	C62—C61—H61	120.7
C14—C15—H15	120.0	C57—C62—C61	120.7 (4)
C16—C15—H15	120.0	C57—C62—H62	119.6
C17—C16—C15	119.0 (4)	C61—C62—H62	119.6
C17—C16—H16	120.5	N6—C63—C64	114.8 (3)
C15—C16—H16	120.5	N6—C63—H63A	108.6
C16—C17—C18	122.4 (4)	C64—C63—H63A	108.6
C16—C17—Cl3	118.6 (3)	N6—C63—H63B	108.6
C18—C17—Cl3	119.0 (3)	C64—C63—H63B	108.6
C19—C18—C17	118.3 (4)	H63A—C63—H63B	107.5
C19—C18—H18	120.8	C69—C64—C65	118.5 (3)
C17—C18—H18	120.8	C69—C64—C63	119.9 (3)
C18—C19—C14	120.9 (4)	C65—C64—C63	121.3 (3)
C18—C19—H19	119.5	C64—C65—C66	120.6 (4)
C14—C19—H19	119.5	C64—C65—H65	119.7
N2—C20—C21	114.6 (3)	C66—C65—H65	119.7
N2—C20—H20A	108.6	C67—C66—C65	119.8 (4)
C21—C20—H20A	108.6	C67—C66—H66	120.1
N2—C20—H20B	108.6	C65—C66—H66	120.1
C21—C20—H20B	108.6	C68—C67—C66	119.5 (4)
H20A—C20—H20B	107.6	C68—C67—H67	120.2
C22—C21—C26	118.6 (3)	C66—C67—H67	120.2
C22—C21—C20	121.8 (3)	C67—C68—C69	120.5 (4)
C26—C21—C20	119.4 (3)	C67—C68—H68	119.8
C21—C22—C23	120.5 (4)	C69—C68—H68	119.8
C21—C22—H22	119.7	C68—C69—C64	121.0 (4)
C23—C22—H22	119.7	C68—C69—H69	119.5
C22—C23—C24	120.4 (4)	C64—C69—H69	119.5
C22—C23—H23	119.8	N7—C70—C82	109.7 (3)
C24—C23—H23	119.8	N7—C70—C80	111.6 (3)
C25—C24—C23	119.2 (4)	C82—C70—C80	100.9 (3)
C25—C24—H24	120.4	N7—C70—C46	103.6 (3)
C23—C24—H24	120.4	C82—C70—C46	120.0 (3)
C24—C25—C26	120.4 (4)	C80—C70—C46	111.1 (3)
C24—C25—H25	119.8	N7—C71—C72	101.7 (3)
C26—C25—H25	119.8	N7—C71—H71A	111.4
C25—C26—C21	120.9 (4)	C72—C71—H71A	111.4
C25—C26—H26	119.5	N7—C71—H71B	111.4
C21—C26—H26	119.5	C72—C71—H71B	111.4
N3—C27—C39	109.0 (3)	H71A—C71—H71B	109.3
N3—C27—C37	111.3 (3)	C73—C72—C71	116.0 (3)
C39—C27—C37	100.7 (3)	C73—C72—C46	115.3 (3)
N3—C27—C3	103.5 (3)	C71—C72—C46	103.6 (3)
C39—C27—C3	120.1 (3)	C73—C72—H72	107.2
C37—C27—C3	112.3 (3)	C71—C72—H72	107.2
N3—C28—C29	102.0 (3)	C46—C72—H72	107.2
N3—C28—H28A	111.4	C78—C73—C74	117.0 (4)
C29—C28—H28A	111.4	C78—C73—C72	120.4 (3)
N3—C28—H28B	111.4	C74—C73—C72	122.5 (4)
C29—C28—H28B	111.4	C73—C74—C75	121.9 (4)
H28A—C28—H28B	109.2	C73—C74—H74	119.1
C30—C29—C28	116.5 (3)	C75—C74—H74	119.1
C30—C29—C3	115.4 (3)	C76—C75—C74	119.1 (4)
C28—C29—C3	103.4 (3)	C76—C75—H75	120.4
C30—C29—H29	107.0	C74—C75—H75	120.4
C28—C29—H29	107.0	C75—C76—C77	121.1 (4)
C3—C29—H29	107.0	C75—C76—Cl8	119.9 (3)
C31—C30—C35	117.9 (4)	C77—C76—Cl8	119.0 (4)
C31—C30—C29	122.3 (4)	C78—C77—C76	118.8 (4)
C35—C30—C29	119.7 (3)	C78—C77—H77	120.6
C30—C31—C32	121.2 (4)	C76—C77—H77	120.6
C30—C31—H31	119.4	C77—C78—C73	122.1 (4)
C32—C31—H31	119.4	C77—C78—H78	119.0
C33—C32—C31	119.7 (4)	C73—C78—H78	119.0
C33—C32—H32	120.1	N7—C79—H79A	109.5
C31—C32—H32	120.1	N7—C79—H79B	109.5
C34—C33—C32	120.5 (4)	H79A—C79—H79B	109.5
C34—C33—Cl4	119.6 (3)	N7—C79—H79C	109.5
C32—C33—Cl4	119.9 (3)	H79A—C79—H79C	109.5
C33—C34—C35	119.2 (4)	H79B—C79—H79C	109.5
C33—C34—H34	120.4	O6—C80—N8	125.3 (3)
C35—C34—H34	120.4	O6—C80—C70	126.5 (3)
C34—C35—C30	121.4 (4)	N8—C80—C70	107.9 (3)
C34—C35—H35	119.3	C86—C81—C82	122.9 (3)
C30—C35—H35	119.3	C86—C81—N8	127.7 (3)
N3—C36—H36A	109.5	C82—C81—N8	109.3 (3)
N3—C36—H36B	109.5	C83—C82—C81	119.0 (3)
H36A—C36—H36B	109.5	C83—C82—C70	130.8 (3)
N3—C36—H36C	109.5	C81—C82—C70	109.4 (3)
H36A—C36—H36C	109.5	C84—C83—C82	119.3 (3)
H36B—C36—H36C	109.5	C84—C83—H83	120.4
O3—C37—N4	125.1 (3)	C82—C83—H83	120.4
O3—C37—C27	126.2 (3)	C83—C84—C85	120.6 (4)
N4—C37—C27	108.4 (3)	C83—C84—H84	119.7
C43—C38—N4	127.4 (3)	C85—C84—H84	119.7
C43—C38—C39	122.7 (3)	C86—C85—C84	120.7 (4)
N4—C38—C39	109.7 (3)	C86—C85—H85	119.6
C40—C39—C38	118.7 (3)	C84—C85—H85	119.6
C40—C39—C27	131.4 (3)	C81—C86—C85	117.4 (3)
C38—C39—C27	109.1 (3)	C81—C86—H86	121.3
C39—C40—C41	119.2 (3)	C85—C86—H86	121.3
			
C7—N1—O1—C1	−8.1 (4)	C41—C42—C43—C38	−1.2 (6)
C50—N5—O4—C44	−5.8 (4)	N5—O4—C44—C48	133.0 (3)
N1—O1—C1—C5	136.3 (3)	N5—O4—C44—C45	−105.4 (3)
N1—O1—C1—C6	11.4 (3)	N5—O4—C44—C49	8.7 (3)
N1—O1—C1—C2	−102.2 (3)	O4—C44—C45—O5	61.8 (4)
O1—C1—C2—O2	61.5 (4)	C48—C44—C45—O5	176.5 (3)
C5—C1—C2—O2	176.1 (3)	C49—C44—C45—O5	−48.1 (4)
C6—C1—C2—O2	−48.7 (4)	O4—C44—C45—C46	−115.2 (3)
O1—C1—C2—C3	−115.6 (3)	C48—C44—C45—C46	−0.6 (5)
C5—C1—C2—C3	−0.9 (5)	C49—C44—C45—C46	134.9 (3)
C6—C1—C2—C3	134.3 (3)	O5—C45—C46—C47	−158.2 (3)
O2—C2—C3—C4	−159.0 (3)	C44—C45—C46—C47	18.6 (4)
C1—C2—C3—C4	17.8 (4)	O5—C45—C46—C72	−33.4 (5)
O2—C2—C3—C29	−34.1 (5)	C44—C45—C46—C72	143.5 (3)
C1—C2—C3—C29	142.8 (3)	O5—C45—C46—C70	78.9 (4)
O2—C2—C3—C27	79.2 (4)	C44—C45—C46—C70	−104.2 (3)
C1—C2—C3—C27	−104.0 (3)	C48—N6—C47—C46	81.0 (3)
C5—N2—C4—C3	81.5 (3)	C63—N6—C47—C46	−158.1 (3)
C20—N2—C4—C3	−158.5 (3)	C45—C46—C47—N6	−57.0 (3)
C2—C3—C4—N2	−56.2 (3)	C72—C46—C47—N6	179.9 (3)
C29—C3—C4—N2	−179.9 (3)	C70—C46—C47—N6	63.7 (3)
C27—C3—C4—N2	63.7 (3)	C47—N6—C48—C44	−58.4 (4)
C4—N2—C5—C1	−60.1 (4)	C63—N6—C48—C44	−179.5 (3)
C20—N2—C5—C1	179.1 (3)	O4—C44—C48—N6	132.4 (3)
O1—C1—C5—N2	133.9 (3)	C45—C44—C48—N6	19.4 (4)
C6—C1—C5—N2	−110.1 (3)	C49—C44—C48—N6	−112.4 (3)
C2—C1—C5—N2	21.0 (4)	O4—C44—C49—C57	114.6 (3)
O1—C1—C6—C14	111.3 (3)	C48—C44—C49—C57	−1.7 (5)
C5—C1—C6—C14	−5.5 (4)	C45—C44—C49—C57	−136.2 (3)
C2—C1—C6—C14	−139.6 (3)	O4—C44—C49—C50	−7.8 (3)
O1—C1—C6—C7	−9.8 (3)	C48—C44—C49—C50	−124.1 (3)
C5—C1—C6—C7	−126.6 (3)	C45—C44—C49—C50	101.4 (3)
C2—C1—C6—C7	99.3 (3)	O4—N5—C50—C51	−173.0 (3)
O1—N1—C7—C8	−172.0 (3)	O4—N5—C50—C49	0.0 (4)
O1—N1—C7—C6	0.9 (4)	C57—C49—C50—N5	−120.1 (3)
C14—C6—C7—N1	−118.8 (3)	C44—C49—C50—N5	5.2 (4)
C1—C6—C7—N1	5.9 (4)	C57—C49—C50—C51	52.8 (4)
C14—C6—C7—C8	53.8 (5)	C44—C49—C50—C51	178.1 (3)
C1—C6—C7—C8	178.5 (3)	N5—C50—C51—C56	−130.9 (4)
N1—C7—C8—C13	−136.2 (4)	C49—C50—C51—C56	56.7 (5)
C6—C7—C8—C13	51.6 (5)	N5—C50—C51—C52	52.5 (5)
N1—C7—C8—C9	49.1 (5)	C49—C50—C51—C52	−119.9 (4)
C6—C7—C8—C9	−123.2 (4)	C56—C51—C52—C53	−3.1 (5)
C13—C8—C9—C10	−2.8 (5)	C50—C51—C52—C53	173.8 (3)
C7—C8—C9—C10	172.3 (3)	C56—C51—C52—Cl5	176.9 (3)
C13—C8—C9—Cl1	179.5 (3)	C50—C51—C52—Cl5	−6.2 (5)
C7—C8—C9—Cl1	−5.4 (5)	C51—C52—C53—C54	−0.5 (6)
C8—C9—C10—C11	3.3 (6)	Cl5—C52—C53—C54	179.5 (3)
Cl1—C9—C10—C11	−178.9 (3)	C52—C53—C54—C55	3.9 (6)
C9—C10—C11—C12	−1.1 (6)	C53—C54—C55—C56	−3.4 (6)
C10—C11—C12—C13	−1.4 (6)	C54—C55—C56—C51	−0.5 (6)
C11—C12—C13—C8	2.0 (5)	C54—C55—C56—Cl6	177.3 (3)
C11—C12—C13—Cl2	−180.0 (3)	C52—C51—C56—C55	3.6 (5)
C9—C8—C13—C12	0.1 (5)	C50—C51—C56—C55	−173.2 (3)
C7—C8—C13—C12	−175.0 (3)	C52—C51—C56—Cl6	−174.2 (3)
C9—C8—C13—Cl2	−177.9 (3)	C50—C51—C56—Cl6	9.0 (5)
C7—C8—C13—Cl2	7.0 (5)	C50—C49—C57—C62	37.4 (4)
C7—C6—C14—C19	38.1 (4)	C44—C49—C57—C62	−78.0 (4)
C1—C6—C14—C19	−76.8 (4)	C50—C49—C57—C58	−146.2 (3)
C7—C6—C14—C15	−143.4 (3)	C44—C49—C57—C58	98.4 (4)
C1—C6—C14—C15	101.7 (4)	C62—C57—C58—C59	2.5 (5)
C19—C14—C15—C16	3.3 (5)	C49—C57—C58—C59	−173.9 (3)
C6—C14—C15—C16	−175.2 (3)	C57—C58—C59—C60	−0.9 (6)
C14—C15—C16—C17	−1.3 (6)	C58—C59—C60—C61	−1.7 (6)
C15—C16—C17—C18	−1.3 (7)	C58—C59—C60—Cl7	176.2 (3)
C15—C16—C17—Cl3	177.0 (3)	C59—C60—C61—C62	2.6 (6)
C16—C17—C18—C19	1.6 (7)	Cl7—C60—C61—C62	−175.4 (3)
Cl3—C17—C18—C19	−176.6 (3)	C58—C57—C62—C61	−1.6 (5)
C17—C18—C19—C14	0.6 (6)	C49—C57—C62—C61	174.7 (3)
C15—C14—C19—C18	−3.0 (5)	C60—C61—C62—C57	−0.9 (6)
C6—C14—C19—C18	175.5 (3)	C48—N6—C63—C64	−69.4 (4)
C4—N2—C20—C21	169.5 (3)	C47—N6—C63—C64	171.4 (3)
C5—N2—C20—C21	−72.1 (4)	N6—C63—C64—C69	147.5 (3)
N2—C20—C21—C22	−36.1 (5)	N6—C63—C64—C65	−38.7 (5)
N2—C20—C21—C26	149.8 (3)	C69—C64—C65—C66	0.7 (6)
C26—C21—C22—C23	1.6 (6)	C63—C64—C65—C66	−173.2 (4)
C20—C21—C22—C23	−172.5 (4)	C64—C65—C66—C67	0.6 (7)
C21—C22—C23—C24	0.0 (7)	C65—C66—C67—C68	−0.7 (7)
C22—C23—C24—C25	−1.1 (7)	C66—C67—C68—C69	−0.7 (6)
C23—C24—C25—C26	0.6 (6)	C67—C68—C69—C64	2.1 (6)
C24—C25—C26—C21	1.0 (6)	C65—C64—C69—C68	−2.1 (5)
C22—C21—C26—C25	−2.1 (5)	C63—C64—C69—C68	172.0 (4)
C20—C21—C26—C25	172.1 (3)	C71—N7—C70—C82	−163.8 (3)
C28—N3—C27—C39	−163.8 (3)	C79—N7—C70—C82	66.9 (4)
C36—N3—C27—C39	67.4 (4)	C71—N7—C70—C80	85.2 (3)
C28—N3—C27—C37	86.0 (3)	C79—N7—C70—C80	−44.1 (4)
C36—N3—C27—C37	−42.8 (4)	C71—N7—C70—C46	−34.5 (3)
C28—N3—C27—C3	−34.8 (3)	C79—N7—C70—C46	−163.7 (3)
C36—N3—C27—C3	−163.6 (3)	C45—C46—C70—N7	−109.9 (3)
C2—C3—C27—N3	−110.2 (3)	C47—C46—C70—N7	130.3 (3)
C4—C3—C27—N3	130.7 (3)	C72—C46—C70—N7	7.6 (3)
C29—C3—C27—N3	8.3 (3)	C45—C46—C70—C82	12.9 (4)
C2—C3—C27—C39	11.6 (4)	C47—C46—C70—C82	−107.0 (3)
C4—C3—C27—C39	−107.5 (3)	C72—C46—C70—C82	130.4 (3)
C29—C3—C27—C39	130.1 (3)	C45—C46—C70—C80	130.2 (3)
C2—C3—C27—C37	129.7 (3)	C47—C46—C70—C80	10.3 (4)
C4—C3—C27—C37	10.6 (4)	C72—C46—C70—C80	−112.3 (3)
C29—C3—C27—C37	−111.8 (3)	C70—N7—C71—C72	47.9 (3)
C36—N3—C28—C29	177.6 (3)	C79—N7—C71—C72	176.8 (3)
C27—N3—C28—C29	48.0 (3)	N7—C71—C72—C73	−167.9 (3)
N3—C28—C29—C30	−168.2 (3)	N7—C71—C72—C46	−40.6 (3)
N3—C28—C29—C3	−40.4 (3)	C45—C46—C72—C73	−96.1 (3)
C2—C3—C29—C30	−95.6 (3)	C47—C46—C72—C73	25.4 (4)
C4—C3—C29—C30	26.0 (4)	C70—C46—C72—C73	147.5 (3)
C27—C3—C29—C30	147.5 (3)	C45—C46—C72—C71	136.2 (3)
C2—C3—C29—C28	136.0 (3)	C47—C46—C72—C71	−102.4 (3)
C4—C3—C29—C28	−102.4 (3)	C70—C46—C72—C71	19.7 (3)
C27—C3—C29—C28	19.1 (3)	C71—C72—C73—C78	−172.6 (3)
C28—C29—C30—C31	6.5 (5)	C46—C72—C73—C78	66.1 (4)
C3—C29—C30—C31	−115.1 (4)	C71—C72—C73—C74	7.7 (5)
C28—C29—C30—C35	−172.0 (3)	C46—C72—C73—C74	−113.6 (4)
C3—C29—C30—C35	66.4 (4)	C78—C73—C74—C75	−1.3 (6)
C35—C30—C31—C32	−2.4 (6)	C72—C73—C74—C75	178.4 (4)
C29—C30—C31—C32	179.1 (3)	C73—C74—C75—C76	0.2 (6)
C30—C31—C32—C33	0.7 (6)	C74—C75—C76—C77	0.8 (6)
C31—C32—C33—C34	0.5 (6)	C74—C75—C76—Cl8	−179.3 (3)
C31—C32—C33—Cl4	−179.5 (3)	C75—C76—C77—C78	−0.6 (6)
C32—C33—C34—C35	0.1 (6)	Cl8—C76—C77—C78	179.5 (3)
Cl4—C33—C34—C35	−179.9 (3)	C76—C77—C78—C73	−0.7 (6)
C33—C34—C35—C30	−1.8 (6)	C74—C73—C78—C77	1.6 (6)
C31—C30—C35—C34	3.0 (6)	C72—C73—C78—C77	−178.1 (3)
C29—C30—C35—C34	−178.5 (3)	C81—N8—C80—O6	174.9 (4)
C38—N4—C37—O3	175.4 (4)	C81—N8—C80—C70	1.3 (4)
C38—N4—C37—C27	0.2 (4)	N7—C70—C80—O6	−59.4 (5)
N3—C27—C37—O3	−60.6 (5)	C82—C70—C80—O6	−175.9 (4)
C39—C27—C37—O3	−176.0 (4)	C46—C70—C80—O6	55.7 (5)
C3—C27—C37—O3	54.9 (5)	N7—C70—C80—N8	114.2 (3)
N3—C27—C37—N4	114.5 (3)	C82—C70—C80—N8	−2.3 (4)
C39—C27—C37—N4	−0.9 (4)	C46—C70—C80—N8	−130.7 (3)
C3—C27—C37—N4	−130.0 (3)	C80—N8—C81—C86	−174.9 (4)
C37—N4—C38—C43	−175.1 (4)	C80—N8—C81—C82	0.4 (5)
C37—N4—C38—C39	0.6 (4)	C86—C81—C82—C83	3.4 (6)
C43—C38—C39—C40	4.1 (6)	N8—C81—C82—C83	−172.3 (3)
N4—C38—C39—C40	−171.9 (3)	C86—C81—C82—C70	173.6 (3)
C43—C38—C39—C27	174.7 (3)	N8—C81—C82—C70	−2.0 (4)
N4—C38—C39—C27	−1.2 (4)	N7—C70—C82—C83	53.4 (5)
N3—C27—C39—C40	53.2 (5)	C80—C70—C82—C83	171.3 (4)
C37—C27—C39—C40	170.3 (4)	C46—C70—C82—C83	−66.3 (5)
C3—C27—C39—C40	−65.9 (5)	N7—C70—C82—C81	−115.3 (3)
N3—C27—C39—C38	−115.8 (3)	C80—C70—C82—C81	2.6 (4)
C37—C27—C39—C38	1.3 (4)	C46—C70—C82—C81	125.0 (3)
C3—C27—C39—C38	125.1 (3)	C81—C82—C83—C84	−3.7 (5)
C38—C39—C40—C41	−3.8 (5)	C70—C82—C83—C84	−171.5 (4)
C27—C39—C40—C41	−172.0 (4)	C82—C83—C84—C85	1.1 (6)
C39—C40—C41—C42	1.3 (6)	C83—C84—C85—C86	2.0 (6)
C40—C41—C42—C43	1.3 (6)	C82—C81—C86—C85	−0.3 (6)
N4—C38—C43—C42	173.7 (4)	N8—C81—C86—C85	174.5 (4)
C39—C38—C43—C42	−1.5 (6)	C84—C85—C86—C81	−2.4 (6)

### Hydrogen-bond geometry (Å, °)

**Table:**

*D*—H···*A*	*D*—H	H···*A*	*D*···*A*	*D*—H···*A*
N4—H4···O6^i^	0.87 (5)	1.94 (5)	2.804 (4)	176 (4)
N8—H8···O3^ii^	0.68 (5)	2.14 (5)	2.808 (4)	173 (5)

Symmetry codes: (i) −*x*+3/2, *y*, *z*−1/2; (ii) −*x*+3/2, *y*, *z*+1/2.
